# Prediction of Technical Difficulties in Laparoscopic Splenectomy and Analysis of Risk Factors for Postoperative Complications in 468 Cases

**DOI:** 10.3390/jcm7120547

**Published:** 2018-12-14

**Authors:** Michał Wysocki, Dorota Radkowiak, Anna Zychowicz, Mateusz Rubinkiewicz, Jan Kulawik, Piotr Major, Michał Pędziwiatr, Andrzej Budzyński

**Affiliations:** 1 2nd Department of General Surgery, Jagiellonian University Medical College, 31-501 Kraków, Poland; michal92wysocki@gmail.com (M.W.); dradkowiak@gmail.com (D.R.); anna.zychowicz@onet.pl (A.Z.); mateusz.rubinkiewicz@uj.edu.pl (M.R.); jankula@poczta.onet.pl (J.K.); michal.pedziwiatr@uj.edu.pl (M.P.); andrzej.budzynski@uj.edu.pl (A.B.); 2 Centre for Research, Training and Innovation in Surgery (CERTAIN Surgery), 31-501 Krakow, Poland

**Keywords:** laparoscopic splenectomy, intraoperative difficulties, hemorrhage, perioperative complications, surgical education

## Abstract

Prediction of intraoperative difficulties may be helpful in planning surgery; however, few studies explored this issue in laparoscopic splenectomy (LS). We performed retrospective analysis of consecutive 468 patients undergoing LS from 1998 to 2017 (295 women; median age 47 years). The patients were divided into difficult LS and control groups. The inclusion criteria for difficult LS were operative time ≥mean + 2SD; intraoperative blood loss ≥500 mL, intraoperative adverse events (IAE), conversion. Primary outcomes were risk factors for difficult splenectomy and secondary outcomes for perioperative morbidity. Fifty-six patients were included in the difficult LS group (12%). Spleens ≥19 cm and higher participation of younger surgeons in consecutive years were predictive for difficult splenectomy. Age ≥53 years and diagnosis other than idiopathic thrombocytopenic purpura (ITP) were independent risk factors of spleen ≥19 cm. The perioperative morbidity was 8.33%; its OR was increased only by blood loss and IAEs. Only blood loss significantly increased serious morbidity. Male sex, spleens ≥19 cm, and IAEs were independent risk factors for intraoperative hemorrhage. Spleen length ≥19 cm was a risk factor for difficult LS and intraoperative hemorrhage. Diagnoses other than ITP in patients aged ≥53 years with ≥19 cm spleens are predictive for intraoperative difficulties and perioperative complications.

## 1. Introduction

Despite controversies regarding who first performed laparoscopic splenectomy (LS) and when, this procedure relatively quickly became very popular. For more than 25 years, LS replaced traditional open splenectomy in most centers [1], and many LS procedures were performed. Numerous studies were followed to explore the identification of patients who may benefit from minimally invasive approaches, and those who would benefit better from the traditional procedure. Norman Halpern observed that “to understand how best to deal with the various aspects of laparoscopic difficulties, it is wise to review what takes place during the uneventful laparoscopy” [2]. Good examples of profound analyses of the risk factors for difficulty, intraoperative adverse events (IAEs), or conversions to the open approach include the Society of American Gastrointestinal and Endoscopic Surgeons (SAGES) Safe Cholecystectomy Program, and the Tokyo Guidelines. The aim was to avoid serious postoperative complications, mainly bile tree injuries. Many risk factors for difficult cholecystectomy were identified, including age, male sex, episodes of acute cholecystitis, obesity, previous abdominal surgery, clinical signs of acute cholecystitis, and certain ultrasonographic findings [3]. Despite our best efforts, we were not able to find studies documenting a similar attempt for LS. This was the inspiration for the current present study. Our objective was to identify predictive factors for difficult LS.

Considering the safety of the procedure, its risk factors for intraoperative difficulties and adverse events must be studied [4]. Based on our 20 years of collective experience and more than 500 laparoscopic operations of the spleen, we believe that the thorough analysis of materials focused on this issue can help to identify important solutions to this problem [5]. The level of difficulty during surgery can be measured by operation time, blood loss, and conversion rate [6]. The definition of difficult operation is based on subjective criteria; however, all surgeons and residents performing this procedure in our high-volume referral center pointed to prolonged operative time, incidence of IAEs, greater than usual blood loss, and need for conversion as important aspects of a difficult procedure. We hypothesized that this situation is more common in male patients, in obese patients, in cases of larger spleen (a usual finding among patients operated for other indications than idiopathic thrombocytopenic purpura (ITP)), in patients with hemorrhagic diathesis, and when LS is performed by younger surgeons. The next important issue is to identify how difficult LS influences the postoperative outcome. Addressing the issue of difficult LS may inspire the establishment of guidelines for safe LS as in the case of laparoscopic cholecystectomy.

The aim of the study was to find possible risk factors associated with difficult LS and analyze its influence on postoperative morbidity.

## 2. Material and Methods

### 2.1. Design

We designed a case–control study based on the retrospective analysis of consecutive patients undergoing laparoscopic total splenectomy at a university hospital (tertiary referral surgical center) from September 1998 to December 2017. The data were collected in a prospective database adhering to the International Society for Pharmacoeconomics and Outcomes Research (ISPOR) Task Force report [7] by all surgeons. All patients were strictly followed up through 30 postoperative days. We included the preoperative and perioperative demographic and clinical data for all cases. The study complies with the STROBE (STrengthening the Reporting of OBservational studies in Epidemiology) guidelines [8].

Inclusion criteria in study population: elective, total laparoscopic splenectomy.

Inclusion criteria for the ‘difficult splenectomy’ group were: operative time ≥mean + 2 standard deviations (SD), intraoperative blood loss ≥500 mL, occurrence of IAE, and need for conversion to open approach.

Exclusion criteria were patients with splenic trauma, initially submitted to open surgery, partial resections and other spleen-preserving procedures, patients in whom hand-assisted LS (HALS) and single-incision LS techniques were used, or incomplete data.

The patients were divided into two groups: patients who underwent operations regarded as difficult were in the difficult LS group and other patients were in the control group.

### 2.2. Outcomes

The primary outcomes are risk factors for difficult LS, while the secondary outcome is determining the relationship between difficult LS and perioperative morbidity, among other risk factors.

### 2.3. Definitions

The length of the spleen is defined as the longest diameter of this organ measured during the abdominal ultrasonography (US) performed by a surgeon just before surgery. The measurement of operative time started from skin incision and ended with closure. The intraoperative blood loss was the amount of blood aspirated in the suction machine. IAEs were defined as any iatrogenic harmful event occurring during the operation, which had not derived from standard course of operation. Intraoperative blood loss ≥500 mL was considered to be a hemorrhage because losing less than one unit of blood (≈500 mL) usually does not negatively condition of patient and does not lead to hypovolemia and hemorrhagic shock [9,10]. Indications for transfusion were hemorrhagic shock, hemoglobin ≤6 g % or 6–8 g % in the case of evidence for limited compensation and risk factors (e.g., coronary heart disease, systolic heart failure, insufficient cerebral vascular flow) or presence of the following symptoms: tachycardia, hypotension, acute coronary ischemia in electrocardiography (ECG), and acidosis [11]. Perioperative morbidity was defined as any complication or deviation from routine postoperative course observed during 30 days after LS (graded with Clavien–Dindo classification [12]).

### 2.4. Operative Technique

Primary choice of the operative access to peritoneal cavity during LS was a laparoscopic 4 port technique. LS was standardized, similarly with technical considerations described by Misiakos et al. [13]. After transection of gastrosplenic ligament and short gastric vessels with various energy devices, extensive lateral mobilization of the spleen was performed. There were two different techniques of control of splenic vessels. At the beginning of our experience we used, in all cases, a technique referred to as ‘vessels first’, described elsewhere [14,15]. In this technique, the main trunks of splenic artery and vein were identified at the level of pancreatic body/tail, isolated, clipped, and transected. Then, the entire splenic hilum, including branches of all vessels, perivascular fat, and lymphatic tissue, were dissected away in one block from the pancreatic tail, and removed together with the spleen. Later, a different technique referred to as ‘hilar transection’ was introduced, where main, nominate splenic vessels were not isolated. The entire splenic hilum was transected, as close as possible to splenic parenchyma with the new generation of energy devices—Ligasure^®^ (Covidien) and, later, Thunderbeat^®^ (Olympus). Occasionally, some clips are put on larger branches of vessels in the hilum, and the decision about its use belongs to the operating surgeon.

### 2.5. Ethics

This study was conducted according to the Report of the ISPOR Task Force on Retrospective Databases [7]. All procedures followed the ethical standards of the responsible committees on human experimentation (institutional and national) and the 2013 Fortaleza revision of the 1975 Declaration of Helsinki. The study was approved by an independent ethics committee at Jagiellonian University, Krakow, Poland (approval number: 1072/6120/160/2017). Informed consent for the surgical treatment was obtained from all patients before the procedure.

### 2.6. Statistical Analysis

All data were analyzed with Statistica software (version 13.0; StatSoft, Tulsa, OK, USA). The results are presented as mean ± SD, median, and interquartile range (IQR), when appropriate. The study of categorical variables used the Pearson’s chi-square test and chi-square with Yates’ correction when appropriate. The Shapiro–Wilk test was used to check for normal distribution of data. Quantitative data were analyzed with Student’s *t*-test (for normally distributed data) and the Mann–Whitney *U*-test (for nonnormally distributed data). Finally, univariate and multivariate logistic regression analyses were built to identify risk factors for primary and secondary outcomes. Receiver operating characteristic (ROC) curves were used for setting cut-off points. The results were considered statistically significant when their *p*-value was found to be less than 0.05.

### 2.7. Material

In total, 468 LS were included in the study. The study group consisted of 295 (63%) women and 173 (37%) men. Their median age was 47 years (15–84 years). Their median preoperative body mass index was 25.89 (22.94–29.32). The indications for splenectomy are presented in Table 1. The most common indication for LS was ITP (253 cases, 54.06%). Lymphoma was the second most common indication (101 cases, 21.58%). The median length of the spleen, according to US, was 14 cm (11–17 cm) (Table 1).

## 3. Results

The median operative time in the entire group was 100 min (70–130 min). The median blood loss was 50 mL (20–120 mL). IAEs occurred in 38 patients (8.12%), and are presented in detail in Table 2, along with the reasons for conversion to open techniques. Conversion was necessary in 14 cases (2.99%). The conversion rate for LS of spleens ≥19 cm was 6.32%, while the conversion rate for LS of spleens <19 cm was 1.28% (*p* = 0.003). Based on our criteria for ‘difficult splenectomy’—operative time ≥mean + 2SD or intraoperative blood loss ≥500 mL or intraoperative adverse events or need for conversion to open approach—56 patients were included in the ‘difficult splenectomy’ group (12%), while 412 (88%) in control group. Using univariate logistic regression model, we sought for risk factors for ‘difficult splenectomy’ (Table 3). Based on our criteria for difficult LS, i.e., operative time ≥mean + 2SD, intraoperative blood loss ≥500 mL, IAEs, or need for conversion to open approach, 56 patients were included in the difficult LS group (12%) while 412 patients (88%) were included in the control group. Using a univariate logistic regression model, we identified risk factors for difficult LS (Table 3). Based on the multivariate logistic regression model, the preoperative length of spleen and year of surgery were found to be predictive for difficult LS. Other factors were significant in univariate models, but were shown to be nonsignificant in the multivariate approach. Using ROC curve analysis, we determined the cut-off point for length of spleen as measured by US that is predictive for difficult LS as 19 cm (area under the curve = 0.742; 95% confidence interval (CI): 0.672–0.812; *p* <0.001). We also analyzed preoperative determinants that can be predictive about spleen ≥19 cm in length as the only significant factor strongly increasing the risk for difficult LS (Table 4). Age ≥53 years (odds ratio (OR) = 2.43; 95% CI: 1.39–4.24) and diagnosis other than ITP (OR = 33.59; 95% CI: 11.92–94.67) were identified as independent prognostic factors of spleen ≥19 cm in length, and indirect for having difficult LS.

We noted perioperative morbidity of 8.33% (39 cases). Perioperative complications are listed in Table 5.

Difficult splenectomy was not related to increase in odds of perioperative morbidity (OR = 1.69; 95% CI: 0.71–4.06). In all tested risk factors, only blood loss and IAEs were shown to significantly increase the odds for perioperative morbidity, but only in univariate logistic regression models. The multivariate logistic regression model revealed that those factors were not independent risk factors for 30-day complications, as presented in Table 6.

We noted 23 cases (4.91%) of serious complications (III–V classes according to Clavien–Dindo classifications). We tested all possible patient- and treatment-related risk factors for serious perioperative morbidity; however, only intraoperative blood loss was shown to significantly increase the ORs for morbidity (OR = 1.01; 95% CI: 1.00–1.02; *p* = 0.029). (Table 7).

Therefore, intraoperative blood loss was both a risk factor for general perioperative morbidity, as well as for serious perioperative complications; thus, what were the risk factors for significant intraoperative blood loss (≥500 mL)? The multivariate logistic regression analysis showed that male sex (OR = 2.97; 95% CI: 1.01–8.68), length of spleen ≥19 cm measured by US (OR = 10.96; 95% CI: 2.24–53.52), and IAEs other than hemorrhage (OR = 22.27; 95% CI: 6.20–80.0), were independent risk factors for intraoperative bleeding (Table 8).

## 4. Discussion

We analyzed of one of the largest single-center outcomes of LS in this study. We addressed the issues of difficult LS to enable surgeons to be better prepared preoperatively before performing the procedure. The length of spleen ≥19 cm, as measured by US, was an independent risk factor, and increased the odds for difficult LS. Despite the surgeons’ sweat and tears during the procedures, difficult LS did not increase perioperative morbidity. We found intraoperative blood loss to be both an independent risk factor for general perioperative morbidity, as well as for serious perioperative complications of LS. The multivariate logistic regression analysis for independent risk factors for intraoperative hemorrhage revealed that male sex tripled the chances for its occurrence, length of spleen ≥19 cm measured with US increased odds 11 times and, finally, IAEs other than hemorrhage resulted in a 22.27-times increase in the odds.

Unfortunately, we could not find any studies analyzing difficult LS. In case of LS, portal hypertension from liver cirrhosis, severe uncorrected coagulopathy, and massive splenomegaly are considered as contraindications for the laparoscopic approach [16]. Massive splenomegaly (defined as a maximum spleen diameter exceeding 25 cm, an estimated spleen volume exceeding 1000 mL, or both) is described by many researchers as a leading cause of conversion to open techniques, or even as a contraindication to LS because of the limited abdominal working space, with an increased risk of bleeding and difficulty retrieving the spleen [16,17,18]. We also found that spleen ≥19 cm in length is associated significantly with higher difficulty of LS. Indirectly, we proved that age ≥53 years and diagnosis other than ITP were identified as independent prognostic factors of spleen ≥19 cm in length. Thus, higher risk of bleeding, rate of IAEs, and conversion could be expected in older patients and indications other than ITP. However, adequately used HALS can be an alternative to full conversion, although it is classified by some as laparoscopy [19,20]. Somasundaram suggests that only very large spleens (larger than 27 cm) and portal hypertension can be regarded as a relative contraindication to laparoscopy [21]. The European Association for Endoscopic Surgery (EAES) defined splenomegaly as a long axis exceeding 15 cm, and massive splenomegaly as a long axis exceeding 20 cm. The EAES recommend HALS or open splenectomy in the case of massive splenomegaly [6]. However, most studies clearly demonstrate the benefits of LS even in the case of massively enlarged spleens. This was very well shown when Bai et al. compared LS to open splenectomy [22]. Throughout our entire experience, we believe that beginning each procedure laparoscopically is beneficial. In the case of serious obstacles during procedure, HALS was used as alternative to conversion; however, HALS cases were excluded from our analysis. In case of spleens ≥19 cm, 6.32% out of 95 cases were converted, while the conversion rate in LS of spleens <19 cm was 1.28%. Thus, based on our experience, we agree with the statements in the EAES recommendations and more recent publications by Misiakos et al. [13].

Regardless of its intraoperative difficulties, we believe that LS is a safe procedure with a low risk of complications and incidental mortality. A meta-analysis by Bai et al. showed much better results concerning mortality and morbidity for LS when compared to open operation [22]. Two patients died postoperatively in our series. This mortality of 0.4% was rather low compared with the ranges presented in the literature (0%–4%) [17,22,23]. Considering perioperative morbidity, which was also low (8.33%), only 23 patients (4.9%) out of 468 suffered from serious (Clavien–Dindo III–V) complications. This finding does not differ much from the data presented in the literature, where postoperative morbidity ranges from 0% to 37.5% [17,23]. Surprisingly, in our series, the difficult LS did not translate directly into perioperative morbidity. Wang et al. determined that high comorbidity index and large spleen are independent risk factors for serious perioperative comorbidity in their analysis of 302 LS cases [23]. Nevertheless, all cited works stated that the most common and worrisome complication of LS is intraabdominal hemorrhage [22,24,25,26]. We have also shown that out of all the possible reasons for regarding splenectomy difficult, only intraoperative blood loss is a direct, independent risk factor for both general and serious perioperative morbidity. In a multivariate analysis, massive intraoperative bleeding (≥500 mL blood loss) was related to male patients (OR = 2.97; 95% CI: 1.01–8.68), enlarged spleens (length of spleen ≥19 cm measured with US, OR = 10.96; 95% CI: 2.24–53.52), and occurrence of IAEs (excluding hemorrhage, OR = 22.27; 95% CI: 6.20–80.0). The combination of those three risk factors is highly, but indirectly, associated with perioperative complications. Therefore, those patients require careful postoperative observation and additional attention.

Another interesting factor is that chances for difficult LS slightly increased with the passage of time. This is because the operations initially carried out by attending surgeons were later performed by junior surgeons and residents. All of them had prior experience in laparoscopic surgery, but they were on the learning curve of LS. The decision to allow trainees to perform LS was left to the senior attending surgeons of our facility.

Nevertheless, further studies are needed to clearly identify the preoperative and intraoperative risk factors. Future studies of difficult LS would help to create safety guidelines such as those developed by SAGES for laparoscopic cholecystectomy. We would like to also endorse the vessels first and HALS techniques to address some of these intraoperative difficulties, such as an enlarged spleen. However, this discussion is ongoing.

Our study is limited by typical factors for a single-center retrospective analysis. However, this is one of the largest published groups of patients undergoing LS in a referral center, where many procedures are performed annually. Therefore, our results cannot be directly transferred to other hospitals. The study material consists of operations performed by five surgical teams, not a single surgeon. Another limitation is the fact that we did not include potential postoperative complications that might have occurred later than 30 days after discharge in this analysis. These complications could certainly influence the overall complication rate. The number of explanatory variables may be excessive for evaluating the effect of each variable because the performance of logistic regression analysis decreases with the increase in variables per outcome.

## 5. Conclusions

We showed that spleen length ≥19 cm and higher participation of younger surgeons in consecutive years increased the possibility of difficult LS and intraoperative hemorrhage. However, this did not seem to translate to perioperative morbidity related to diagnosis other than ITP, age ≥53 years, and splenomegaly ≥19 cm.

## Figures and Tables

**Table 1:** Indications for LS.

Indication	Number (%)	Median US Size of the Spleen, cm (IQR)
ITP	253 (53.26%)	11 (10–13)
Lymphoma	101 (21.58%)	19 (17–23)
Spherocytosis	28 (5.98%)	18 (15–20)
Autoimmune anemia	21 (4.49%)	15 (14–17)
Tumor	20 (4.27%)	14 (11–15.5)
Splenomegaly	22 (4.70%)	18 (16–20)
Leukemia	8 (1.71%)	17.5 (16–21)
Other	15 (3.21%)	15 (12–20)
Total	468 (100%)	14 (11–17)

LS—laparoscopic splenectomy, ITP—idiopathic thrombocytopenia, US—ultrasonography, IQR—interquartile range.

**Table 2:** Intraoperative adverse events and reasons for conversions.

**Intraoperative Adverse Events**		**Number of Cases**
Accidental injury of intraabdominal organ	Stomach	2 (0.43%)
	Splenic flexure	2 (0.43%)
	Diaphragm (sutured without chest tube)	1 (0.21%)
	Small bowel	1 (0.21%)
	Pancreatic tail	1 (0.21%)
	Bladder (Pfannenstiel incision)	1 (0.21%)
Massive bleeding	Managed laparoscopically	20 (4.27%)
	Requiring conversion	5 (1.07%)
Rupture of the retrieval bag		5 (1.07%)
Total		38 (8.12%)
**Reasons for Conversions**		**Number of Cases**
Bleeding		5 (1.07%)
Technical problems		4 (0.85%)
Infiltration of surrounding structures		3 (0.64%)
Spleen’s friability		1 (0.21%)
Size of the spleen		1 (0.21%)
Total		14 (2.99%)

**Table 3:** Risk factors for difficult splenectomy.

Parameter	Difficult Splenectomy	Control Group	OR	95% CI	*p*-Value
*n* (%)	56 (12%)	412 (88%)	n/a	n/a	n/a
**Univariate**					
Age, median years (IQR)	51 (35–62)	46 (28–58)	1.01	1.00–1.03	0.150
Males/Females, *n* (%)	29/27 (52%/48%)	144/268 (35%/65%)	0.50	0.28–0.88	0.016
BMI, median kg/m^2^ (IQR)	26.43 (23.66–28.04)	25.83 (22.55–29.38)	0.99	0.91–1.08	0.790
ITP vs. other, *n* (%)	18/38 (32%/68%)	235/177 (57%/43%)	2.80	1.54–5.08	0.001
US length of spleen, median cm (IQR)	19 (14–22)	13 (11–17)	1.14	1.09–1.20	<0.001
US length of spleen ≥19 cm, *n* (%)	28 (54%)	60 (15%)	6.59	3.57–12.16	<0.001
Lowest preoperative platelets, median 10^3^/mm^3^ (IQR)	8 (6–23)	8 (3–15)	1.00	0.98–1.03	0.772
Preoperative platelets, median 10^3^/mm^3^ (IQR)	55 (31–64)	83 (50–120)	1.00	0.99–1.03	0.268
Need for extra method of preparing patients for LS, *n* (%)	20 (36%)	252 (61%)	0.35	0.20–0.63	<0.001
Accessory spleen, *n* (%)	4 (7.14%)	69 (16.75%)	0.38	0.12–1.10	0.073
Technique, n (%) -vessels first -hilar transection	19 (34%) 37 (66%)	150 (36%) 262 (64%)	1.15	0.58–2.25	0.694
Year of surgery			1.07	1.01–3.90	0.027
**Multivariate**					
Male sex			0.58	0.31–1.10	0.094
Diagnosis other than ITP			0.98	0.35–2.75	0.965
US length of spleen ≥19 cm			5.46	2.45–12.18	**<0.001**
Need for extra method of preparing patients for LS			0.87	0.33–2.27	0.768
Year of surgery			1.10	1.10–1.19	**0.011**

IQR—interquartile range, OR—odds ratio, CI—confidence interval, BMI—body mass index, ITP—idiopathic trombocytopenia, US—ultrasound, LS—laparoscopic splenectomy.

**Table 4:** Risk factors for spleen ≥19 cm in length.

Parameter	≥19 cm	<19 cm	OR	95% CI	*p*-Value
*n* (%)	91 (19%)	377 (81%)	n/a	n/a	n/a
**Univariate**					
Age, median years (IQR)	59 (44–67)	44 (27–56)	1.04	1.03–1.06	<0.001
Age ≥53 years, *n* (%)	60 (66%)	125 (33%)	3.92	2.39–6.41	<0.001
Males/Females, *n* (%)	44/47 (48%/52%)	131/246 (35%/65%)	1.80	1.12–2.88	0.015
BMI, median kg/m^2^ (IQR)	25.52 (23.29–28.04)	26.02 (22.48–29.38)	0.99	0.92–1.07	0.891
Obesity, *n* (%)	11 (12%)	74 (20%)	0.58	0.21–1.63	0.300
ITP vs. other, n (%)	4/87 (4%/96%)	247/130 (66%/34%)	39.98	14.28–111.92	<0.001
Year of surgery			1.06	0.99–1.12	0.052
**Multivariate**					
Age ≥53 years			2.43	1.39–4.24	0.002
Females			0.60	0.34–1.04	0.066
Diagnosis other than ITP			33.59	11.92–94.67	<0.001

IQR—interquartile range, OR—odds ratio, CI—confidence interval, BMI—body mass index, ITP—idiopathic trombocytopenia.

**Table 5:** Perioperative (≤30 days) complications according to Clavien–Dindo grading.

C–D	Complications	Number of Cases
5	Pulmonary embolism (patient death)	1 (0.21%)
	Massive intraoperative blood loss. Reoperation for recurrent hemorrhage (patient death)	1 (0.21%)
4b	Pancreatic fistula with abscess and secondary perforation of splenic flexure. Multiple reoperations. Cardiorespiratory failure (ICU stay)	1 (0.21%)
3b	Postoperative bleeding (relaparoscopy)	6 (1.28%)
	Subphrenic abscess (reoperation)	6 (1.28%)
	Acute pancreatitis (reoperation)	2 (0.43%)
	Perforation of the fundus of the stomach probably due to thermal injury (reoperation on 8th postoperative day)	1 (0.21%)
	Thrombosis of superior mesenteric artery (revascularization)	1 (0.21%)
3a	Subphrenic abscess (percutaneous drainage)	1 (0.21%)
	Port-site bleeding (suture-ligation under local anesthesia)	1 (0.21%)
	Pelvic hematoma (percutaneous drainage)	1 (0.21%)
	Pneumothorax (chest tube)	1 (0.21%)
2	Fever of unknown origin	7 (1.50%)
	Pulmonary infection	2 (0.43%)
	Urinary tract infection	1 (0.21%)
	Superior mesenteric vein thrombosis	1 (0.21%)
	Subphrenic hematoma	1 (0.21%)
1	Subphrenic fluid collection	3 (0.64%)
	Pneumothorax (spontaneous resolution)	1 (0.21%)
	Total	39 (8.33%)

C–D—Clavien–Dindo, ICU—Intensive Care Unit.

**Table 6:** Risk factors for perioperative morbidity.

Parameter	Complicated	Non-Complicated	OR	95% CI	*p*-Value
Difficult splenectomy, *n* (%)	7 (12.50%)	49 (87.50%)	1.69	0.71–4.06	0.234
No difficult splenectomy, *n* (%)	32 (7.77%)	380 (92.23%)			
Age, median years (IQR)	40 (27–57)	47 (29–59)	0.99	0.97–1.01	0.162
Males/Females, *n* (%)	17/22 (44%/56%)	156/273 (36%/64%)	0.74	0.38–1.44	0.372
BMI, median kg/m^2^ (IQR)	27.51 (23.36–32.37)	25.82 (22.94–29.28)	1.06	0.92–1.22	0.442
ITP vs. other, *n* (%)	22/17 (56%)	231/198 (54%)	0.90	0.46–1.75	0.758
US length of spleen, median cm (IQR)	14.5 (12–18)	13.5 (11–17)	1.02	0.96–1.08	0.548
Lowest preoperative platelets, median 10^3^/mm^3^ (IQR)	10 (2.5–21.5)	8 (4–15)	1.00	0.97–1.03	0.963
Preoperative platelets, median 10^3^/mm^3^ (IQR)	69 (38–126)	79.5 (50–120)	1.00	0.99–1.01	0.695
Need for extra method of preparing patients for LS, *n* (%)	23 (58.97%)	249 (58.04%)	1.04	0.52–2.06	0.912
Accessory spleen, *n* (%)	7 (17.95%)	66 (15.38%)	1.20	0.51–2.85	0.673
Technique, *n* (%) -vessels first -hilar transection	17 (44.74%) 21 (55.26%)	147 (35.34%) 269 (64.66%)	0.68	0.234–1.32	0.251
Operative time, median min (IQR)	95 (75–120)	100 (70–130)	0.99	0.98–1.01	0.614
Blood loss, median ml (IQR)	100 (40–175)	50 (20–100)	1.01	1.00–1.02	0.003
Conversion, *n* (%)	1 (2.56%)	13 (3.03%)	0.84	0.11–6.64	0.870
Intraoperative adverse events, *n* (%)	5 (12.82%)	20 (4.66%)	3.01	1.06–8.54	0.038
Year of surgery			0.93	0.87–1.01	0.050
**Multivariate**					
Blood loss ≥500 mL			2.94	0.70–12.27	0.138
Intraoperative adverse events			1.00	0.99–1.02	0.210

IQR—interquartile range, OR—odds ratio, CI—confidence interval, BMI—body mass index, ITP—idiopathic trombocytopenia.

**Table 7:** Risk factors for perioperative serious perioperative morbidity (Clavien–Dindo III–V).

Parameter	Complicated	Non-Complicated	OR	95% CI	*p*-Value
Difficult splenectomy, *n* (%)	5 (8.93%)	51 (91.07%)	0.01	0.003–1.62	0.996
No difficult splenectomy, *n* (%)	18 (4.37%)	394 (95.63%)			
Age, median (IQR)	47 (29–60)	47 (29–59)	0.99	0.97–1.02	0.964
Males/Females, *n* (%)	12/11 (52%/48%)	161/284 (36%/64%)	0.52	0.22–1.21	0.127
BMI, median kg/m^2^ (IQR)	27.51 (18.59–32.37)	25.83 (22.94–29.32)	1.01	0.86–1.19	0.913
ITP vs. other, *n* (%)	13/10 (57%/43%)	240/205 (54%/46%)	0.90	0.39–2.10	0.808
US length of spleen, median cm (IQR)	14.5 (12–17)	14 (11–17)	1.03	0.95–1.11	0.454
Lowest preoperative platelets, median 10^3^/mm^3^ (IQR)	7 (1–12)	8 (4–17)	0.98	0.93–1.03	0.452
Preoperative platelets, median 10^3^/mm^3^ (IQR)	59 (31–102)	79.5 (50–120)	0.99	0.98–1.00	0.246
Need for extra method of preparing patients for LS, *n* (%)	15 (65%)	257 (58%)	1.37	0.57–3.31	0.481
Accessory spleen, *n* (%)	5 (21.74%)	68 (15.28%)	1.54	0.55–4.30	0.408
Technique, *n* (%) -vessels first -hilar transection	7 (30.43%) 16 (69.57%)	157 (36.43%) 274 (63.57%)	1.31	0.53–3.26	0.561
Operative time, median min (IQR)	105 (80–120)	100 (70–130)	0.99	0.99–1.01	0.945
Blood loss, median ml (IQR)	100 (30–200)	50 (20–100)	1.01	1.00–1.02	0.029
Conversion, *n* (%)	0	14 (3.15%)	n/a	n/a	n/a
Intraoperative adverse events, *n* (%)	3 (13.04%)	22 (4.94%)	2.88	0.79–10.48	0.107
Year of surgery			0.97	0.89–1.06	0.475

IQR—interquartile range, OR—odds ratio, CI—confidence interval, BMI—body mass index, ITP—idiopathic trombocytopenia, US—ultrasound.

**Table 8:** Risk factors for intraoperative blood loss ≥500 mL.

Parameter	≥500mL	Control	OR	95% CI	*p*-Value
*n* (%)	25 (5.34%)	443 (94.66%)	n/a	n/a	n/a
**Univariate**					
Age, median (IQR)	54 (40–63)	50 (32–53)	1.01	0.99–1.04	0.373
Males/Females, *n* (%)	15/10 (60%/40%)	156/287 (35%/65%)	2.75	1.19–6.38	0.018
BMI, median kg/m^2^ (IQR)	26.66 (23.66–28.04)	25.98 (22.74–29.39)	0.99	0.90–1.10	0.917
Obesity, *n* (%)	3 (12%)	89 (20%)	0.50	0.11–2.29	0.366
ITP vs. other, *n* (%)	7/18 (28%/72%)	213/230 (48%/52%)	2.39	1.00–5.93	0.044
US length of spleen, median cm (IQR)	20 (17–25)	14 (11–17)	1.24	1.14–1.34	<0.001
US length of spleen ≥19 cm, *n* (%)	17 (68%)	71 (16%)	11.12	4.50–27.49	<0.001
Lowest preoperative platelets, median 10^3^/mm^3^ (IQR)	10 (7–38)	7.5 (3–17)	1.01	0.98–1.04	0.479
Preoperative platelets, median 10^3^/mm^3^ (IQR)	51 (28–63)	84.5 (50–120)	0.99	0.97–1.00	0.064
Need for extra method of preparing patients for LS, *n* (%)	8 (32%)	236 (53%)	0.41	0.17–0.99	0.047
Accessory spleen, *n* (%)	1 (4%)	67 (15%)	0.23	0.03–1.80	0.161
Technique, *n* (%) -vessels first -hilar transection	7 (28%) 18 (72%)	56 (13%) 387 (87%)	0.41	0.14–1.20	0.102
Intraoperative adverse events other than hemorrhage, *n* (%)	12 (48%)	13 (3%)	30.46	10.57–87.76	<0.001
Year of surgery			1.10	0.95–1.28	0.186
**Multivariate**					
Male sex			2.97	1.01–8.68	0.046
Diagnosis other than ITP			1.64	0.11–3.45	0.579
US length of spleen ≥19 cm			10.96	2.24–53.52	0.003
Need for extra method of preparing patients for LS			1.76	0.38–8.27	0.469
Intraoperative adverse events other than hemorrhage			22.27	6.20–80.0	<0.001

IQR—interquartile range, OR—odds ratio, CI—confidence interval, BMI—body mass index, ITP—idiopathic trombocytopenia, US—ultrasound, LS—laparoscopic splenectomy.

## Abbreviations

LS—laparoscopic splenectomy;

IAE—intraoperative adverse events;

ITP—immune trombocytopenic purpura;

SAGES—Society of American Gastrointestinal and Endoscopic Surgeons;

ISPOR—International Society for Pharmacoeconomics and Outcomes Research;

STROBE—Strengthening the Reporting of Observational studies in Epidemiology;

HALS—hand-assisted laparoscopic surgery;

SILS—single incision laparoscopic surgery;

SD—standard deviation;

ECG—electrocardiography;

IQR—interquartile range;

ROC—receiver operating characteristic curve;

BMI—body mass index;

US—ultrasound/ultrasonography;

EAES—European Association for Endoscopic Surgery.
